# Association between superficial surgical site infection and revision after primary total hip arthroplasty: a cohort study of 3,242 patients

**DOI:** 10.2340/17453674.2026.46223

**Published:** 2026-07-08

**Authors:** Christian T POLLMANN, Owen M T THOMAS, Jan Harald M RØTTERUD, Håvard DALE, Mireille W H WULF, Inge SKRÅMM

**Affiliations:** 1 Department of Orthopaedic Surgery, Akershus University Hospital, Lørenskog; 2 Health Services Research Unit, Akershus University Hospital, Lørenskog; 3 Oslo Centre for Biostatistics and Epidemiology, Research Support Services, Oslo University Hospital, Oslo; 4 Norwegian Arthroplasty Register, Department of Orthopaedic Surgery, Haukeland University Hospital, Bergen; 5 Department of Clinical Medicine (K1), University of Bergen, Bergen; 6 Department of Medical Microbiology and Infection Control, Akershus University Hospital, Lørenskog, Norway

## Abstract

**Background and purpose:**

We aimed to evaluate whether a superficial surgical site infection (SSI) after primary total hip arthroplasty (THA) is associated with an increased risk of subsequent revision hip arthroplasty for any cause.

**Methods:**

This is a single-center, observational study based on data from the Norwegian Arthroplasty Register (NAR) and the local infection surveillance program. All patients operated on with a primary THA at our institution from January 1999 through December 2019 were eligible for the study. The risk of revision surgery for any cause was compared between the exposed (superficial SSI) and the unexposed group (no SSI) with frequentist (Cox regression) and Bayesian survival analysis.

**Results:**

3,242 patients were included in the study. Median follow-up in the NAR was 7, minimum follow-up was 4 years. 27 patients were registered with a superficial SSI. A Cox regression analysis adjusted for sex, age, American Society of Anesthesiologists Physical Status (ASA) class, duration of surgery, and year of surgery gave an adjusted hazard ratio of 1.4 (95% confidence interval: 0.3–5.9, P = 0.6) for the risk of revision for any cause in the exposed group (superficial SSI) compared with the unexposed group (no SSI). A Bayesian survival model, adjusted for the same covariates, gave a censored time coefficient of 0.5 (95% credible interval: 0.1–0.9), with a corresponding very low (1%) probability of an increased risk of revision for any cause in the exposed group.

**Conclusion:**

We showed no association between superficial SSI and revision after primary THA. Thus, our data does not support the dogma that superficial SSIs after primary THA do not exist.

Surgical site infection (SSI) after total joint arthroplasty is a serious complication [1]. Both the literature and surveillance systems typically differentiate between superficial and deep SSI [2-4]. This distinction has profound consequences for the treatment approach. That is, deep SSI is usually treated with some type of deep surgical revision including the exchange of some parts of or the whole implant [5], while superficial SSI is treated with either antibiotics alone or in combination with superficial surgical revision.

Evidence is sparse but suggests an increased risk of subsequent major surgery in patients with a superficial SSI after total joint arthroplasty [3,6,7]. This is in line with the belief of many arthroplasty surgeons that superficial SSIs do not really exist but rather represent overlooked deep infections. Consequently, one might advocate for revision surgery in all cases of SSI after total joint arthroplasty irrespective of the supposed depth of the infection.

In this single-center, retrospective study using routinely collected data, we aimed to answer the question of whether a superficial SSI after primary total hip arthroplasty (THA) was associated with increased risk of subsequent revision hip arthroplasty.

## Methods

### Study design

This is a single-center, observational study at Akershus University Hospital (AHUS) based on data from the Norwegian Arthroplasty Register (NAR), the local surveillance system for SSIs, and the electronic hospital records.

The study is reported according to STROBE guidelines.

### Patients and data collection from the Norwegian Arthroplasty Register

All primary and revision THAs in Norway should be reported to the NAR. The unique identification number of each Norwegian citizen is used to link the primary THA to any subsequent revisions. The completeness of the NAR has been shown to be 97% for primary THAs and 93% for revisions [8,9].

All patients operated on with a primary total hip arthroplasty for any indication at AHUS from January 1999 through December 2019 were identified in the NAR. December 31, 2023 was chosen as the end of follow-up in the NAR.

### Data on surgical site infection

In cooperation with the Department of Microbiology and Infection Control, the Department of Orthopedic Surgery at AHUS has surveyed SSIs after primary total hip arthroplasty with 30-day follow-up since 1998. From 2005 onwards this surveillance became part of a national program, the Norwegian Surveillance System for Antibiotic Use and Hospital-Acquired Infections (NOIS). A questionnaire is sent to each patient or, in the case of cognitive impairment or institutionalization, to the primary healthcare provider. If the patient reports an SSI or a suspicion of SSI this is confirmed by a physician on the same questionnaire. In equivocal cases the electronic hospital records are scrutinized, the primary healthcare provider may be contacted, and an orthopedic surgeon (the same surgeon since 1998) is consulted.

Until 2014, cases of superficial and deep SSI were defined according to the American Centers for Disease Control and Prevention [2], while from 2014 and onwards case definitions from the European Centre for Disease Prevention and Control [10] have been applied (Table 1). Concerning SSIs, both definitions are practically identical.

**Table 1:** Surgical site infection: definitions [10]

**Superficial surgical site infection** Infection occurs within 30 days after the operation AND infection involves only skin and subcutaneous tissue of the incision AND at least one of the following: Purulent drainage with or without laboratory confirmation, from the superficial incision Organisms isolated from an aseptically obtained culture of fluid or tissue from the superficial incision At least one of the following signs or symptoms of infection: pain or tenderness, localized swelling, redness, or heat AND superficial incision is deliberately opened by surgeon, unless incision is culture-negative Diagnosis of superficial incisional SSI made by a surgeon or attending physician
**Deep surgical site infection** Infection occurs within 30 days after the operation if no implant is left in place or within one year if implant is in place AND the infection appears to be related to the operation AND infection involves deep soft tissue (e.g., fascia, muscle) of the incision AND at least one of the following: Purulent drainage from the deep incision but not from the organ/space component of the surgical site A deep incision spontaneously dehisces or is deliberately opened by a surgeon when the patient has at least one of the following signs or symptoms: fever (> 38 °C), localized pain, or tenderness, unless incision is culture-negative An abscess or other evidence of infection involving the deep incision is found on direct examination, during reoperation, or by histopathological or radiological examination Diagnosis of deep incisional SSI made by a surgeon or attending physician

The overall completeness of the 30-day follow-up for SSI was 99.3%, ranging from 97.2% to 100% per calendar year.

### Patient inclusion

Patients who were registered with a deep SSI during the first 30 days after the primary THA were excluded.

Patients with bilateral THA during the study period were included only once (for patients without SSI, the first THA was included; for patients with superficial SSI, the side with the superficial SSI was included); for patients with deep SSI, the other side was also excluded. Patients with an incomplete dataset (missing data on American Society of Anesthesiologists Physical Status [ASA] class and/or length of surgery; n = 554) were excluded.

### Statistics

The primary endpoint of this study was revision hip arthroplasty for any cause. The comparison groups were patients with superficial SSI (exposed group) and without SSI (unexposed group) after primary THA. The risk of revision hip arthroplasty for any cause was compared using both frequentist (Cox regression) and Bayesian survival analysis. The time-to-event was calculated from the date of surgery for the primary THA to the date of revision surgery. Censoring mechanisms were death or the end of the study period (December 31, 2023).

For the Bayesian survival analysis, the model was formulated to directly model the time to outcome event as a linear combination of the covariates, with appropriate handling of the censoring, with inference performed in the brms package [11]. Non-informative, Gaussian priors N(0,10^2^) for the regression parameters were used. It is important to note that while the Cox analyses model the hazard ratio of an increase in risk of revision, the Bayesian analyses, motivated by available software implementations, directly model the time-to-event while accounting for censoring. We consequently a priori expect effect sizes in opposite directions between the frequentist and Bayesian analyses, as a larger hazard ratio corresponds to a smaller time-to-event. We present censored time coefficients with 95% credible intervals and corresponding probabilities of an effect on the outcome.

For the Cox models, the proportional hazards assumption was evaluated using Schoenfeld residuals and deemed to be not severely violated. The hypothesis test for proportional hazards was deemed to be inappropriate considering the large size of the data set leading to the failure of any goodness-of-fit test. We present hazard ratios (HR) with 95% confidence intervals (CI) for the exposed (superficial SSI) compared with the unexposed (no SSI) group.

**Figure 1:**
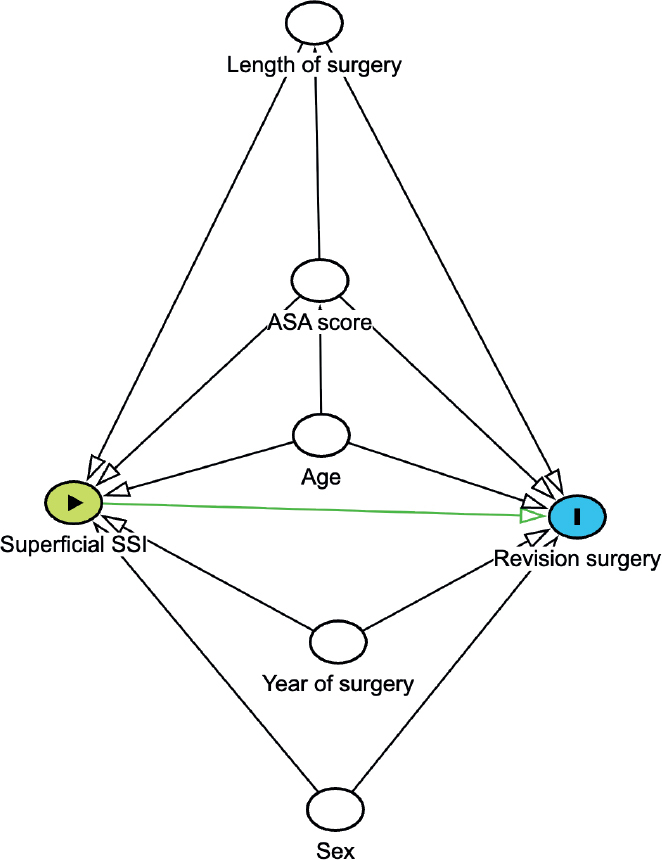
Directed acyclic graph showing the causal model used as a basis for analyzing the association between superficial surgical site infection and revision hip arthroplasty for any cause. SSI: surgical site infection; ASA: American Society of Anesthesiologists. 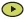 exposure, 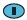 outcome, 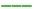 adjusted variable, 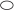 causal path, 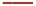 biasing path (none present after adjustment).

We present unadjusted and adjusted analyses. Covariates for the adjusted analyses were chosen employing clinical reasoning. The relationship of these covariates with each other as well as with the exposure variable (superficial SSI) and the primary endpoint (revision hip arthroplasty for any cause) were visualized with a directed acyclic graph (DAG) (Figure 1), which was drawn using DAGitty [12]. In the adjusted analyses, both the Cox models and the Bayesian survival models were adjusted for sex, age, ASA class, duration of surgery, and year of surgery, as motivated by the DAG.

The statistical analyses were performed with R version 4.5.1 (R Foundation for Statistical Computing, Vienna, Austria) [13].

### Sensitivity analysis

As a sensitivity analysis, the competing risk of death (prior to an eventual revision hip arthroplasty) was addressed by analyzing a composite endpoint of death or revision hip arthroplasty for any cause.

### Ethics, use of AI, funding, and disclosures

The study was approved by the Regional Ethics Committee South East C (reference number 664213). The Norwegian Data Inspectorate approved the registration of data in the NAR. Data was collected and handled in accordance with the requirements of the local data protection officer. AI was not used for any part of this study. The study received no funding, and the authors declare no conflicts of interest. Complete disclosure of interest forms according to ICMJE are available on the article page, doi: 10.2340/17453674.2026.46223

## Results

### Patient characteristics

**Figure 2:**
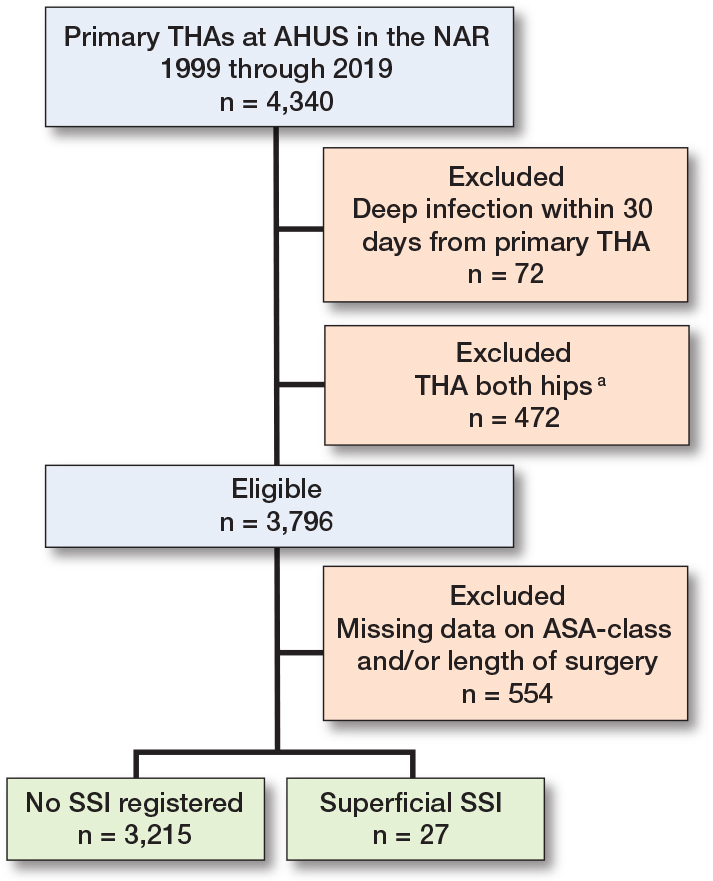
Flowchart of patient inclusion. AHUS: Akershus University Hospital; ASA: American Society of Anesthesiologists. NAR: Norwegian Arthroplasty Register. SSI: surgical site infection; THA: total hip arthroplasty. **^a^** For patients without surgical site infection (SSI), only the first THA was included (n = 452); for patients with superficial SSI, only the side with the superficial SSI was included (n = 7); for patients with deep SSI, the other side was also excluded n = 13).

3,242 patients were included in this study (Figure 2) of which 27 (0.8%) were registered with a superficial SSI. Patient characteristics are presented in Table 2. None of the patients registered with a superficial SSI were treated surgically for this infection.

**Table 2:** Patient characteristics. Values are count (%) unless otherwise specified

Factor	Unexposed (No SSI n = 3,215	Exposed (Superficial SSI) n = 27
Age at primary THA, mean (SD)	69 (11)	71 (10)
Female sex	2,073 (65)	21 (78)
ASA class at primary THA		
1	386 (12)	–
2	1,850 (58)	17 (63)
3	963 (30)	10 (37)
4	16 (0.5)	–
Minutes of surgery, median (IQR)	89 (75–103)	70 (56–100)

SSI: surgical site infection; THA: total hip arthroplasty; SD: standard deviation; ASA: American Society of Anesthesiologists; IQR: interquartile range.

Minimum follow-up was 4 years (end of inclusion December 31, 2019 to end of study December 31, 2023). Median follow-up was ~7 years, comparable in the exposed and the unexposed group (Table 3).

**Table 3:** Patient outcomes. Unadjusted analyses of risk of revision surgery for any cause and of the composite endpoint of death or revision surgery for any cause

Factor	Unexposed (No SSI) n = 3,215	Exposed (Superficial SSI) n = 27
Years of follow-up, median (IQR) **^a^**	6.8 (4.9–9.8)	7.0 (4.8–14.3)
Primary event, n	149	2
Unadjusted HR (CI)	1.3 (0.3 to 5.3)	
Unadjusted Bayesian censored time coefficient (95% credible interval)	–0.8 (–1.2 to –0.4)	
Corresponding probability of increased risk of primary endpoint in exposed group	100%	
Composite endpoint, n	1,000	17
Unadjusted HR composite endpoint (CI)	1.2 (0.7 to 2.0)	
Unadjusted Bayesian censored time coefficient composite endpoint (95% credible interval)	–2.0 (–3.1 to –1.0)	
Corresponding probability of increased risk of composite endpoint in exposed group	100%	

CI: 95% confidence interval; composite endpoint = death or revision; IQR: interquartile range; HR: hazard ratio; SSI: surgical site infection; primary event = revision for any cause;

a Time from primary THA to revision surgery, death, or end of study.

### Risk of revision surgery

**Figure 3:**
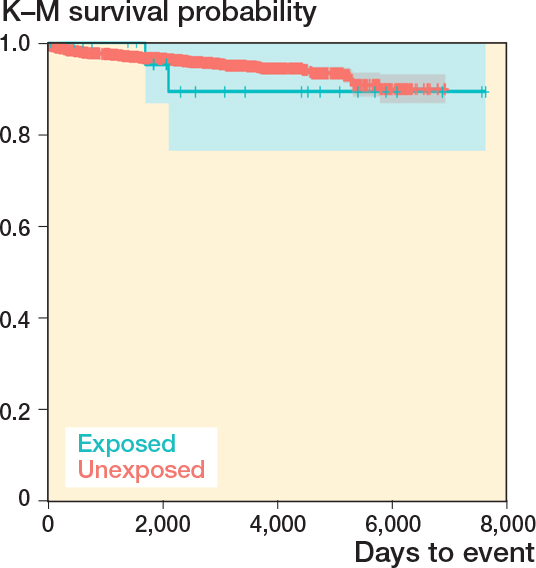
Kaplan–Meier survival curves showing revision-free implant survival for patients without surgical site infection (unexposed group) and patients with superficial surgical site infection (exposed group) after primary total hip arthroplasty. Time-to-event (in days) calculated from date of primary total hip arthroplasty to date of revision surgery. Vertical lines represent censoring events (death or end of study, December 31, 2023). The areas with lighter color represent the 95% confidence intervals. P = 0.7 derived from a log-rank test.

The crude revision rates for any cause were 149 (4.6%) of 3,215 patients without SSI and 2 of 27 (7.4%) patients with superficial SSI. Figure 3 shows the unadjusted Kaplan–Meier survival curves for patients with and without superficial SSI.

Neither of the 2 patients who experienced a superficial SSI was revised due to periprosthetic joint infection (PJI). One patient was revised after 4.7 years due to prosthesis dislocation and the other after 5.8 years due to loosening of the femoral component.

We provide unadjusted hazard ratios in Table 3 to demonstrate the influence of the covariate adjustment (Table 4) and observe that their inclusion has a larger influence on the Bayesian models than the frequentist models.

**Table 4:** Patient outcomes. Adjusted analyses (adjusted for sex, age, ASA class, duration of surgery, and year of surgery) of risk of revision surgery for any cause and of the composite endpoint of death or revision surgery for any cause

Factor	Unexposed (No SSI) n = 3,215	Exposed (Superficial SSI) n = 27
Years of follow-up, median (IQR)**^a^**	6.8 (4.9–9.8)	7.0 (4.8–14.3)
Primary event, n	149	2
Adjusted HR (CI)	1.4 (0.3 to 5.9)	
Adjusted Bayesian censored time coefficient (95% credible interval)	0.5 (0.1 to 0.9)	
Corresponding probability of increased risk of primary endpoint in exposed group	1%	
Composite endpoint, n	1,000	17
Adjusted HR composite endpoint (CI)	1.1 (0.6 to 1.8)	
Adjusted Bayesian censored time coefficient composite endpoint (95% credible interval)	0.4 (–0.3 to 1.0)	
Corresponding probability of increased risk of primary endpoint in exposed group	11%	

For abbreviations, see Table 3.

An adjusted Cox regression analysis gave an HR of 1.4 (CI 0.3–5.9, P = 0.6) for an increase in risk of revision for any cause in the exposed group (superficial SSI).

An adjusted Bayesian survival model gave a censored time coefficient of 0.5 (95% credible interval: 0.1–0.9) with a corresponding very low (1%) probability for an increased risk of revision for any cause in the exposed group (superficial SSI).

### Sensitivity analysis

To address the problem of competing risk between revision surgery and death, we performed a sensitivity analysis on a composite endpoint of revision for any cause or death.

An adjusted Cox regression analysis gave a HR of 1.1 (CI 0.6–1.8, P = 0.8) for an increase in risk of the composite endpoint in the exposed group (superficial SSI).

A Bayesian survival model gave a censored time coefficient of 0.4, albeit with relatively broad 95% credible interval (–0.3 to 1.0), corresponding to an 11% probability that the risk of the composite endpoint is increased in the exposed group (superficial SSI). The broad posterior suggests poor identifiability of the estimate and consequently the posterior summaries should be interpreted carefully.

## Discussion

The aim of this study was to answer the question of whether superficial SSI after primary THA was associated with an increased risk of subsequent revision hip arthroplasty. If most superficial SSIs after primary THA were truly undetected deep SSIs, one would expect an increased risk of revision arthroplasty after superficial SSI. This is not supported by our data, which show a low probability that a superficial SSI after primary THA increases the risk of subsequent revision hip arthroplasty.

The rate of superficial SSIs in our cohort of 0.8% was in the lower range of published rates after primary THA, which range from 0.7% to 9.5% [3,6,14-16]. We were only able to identify 2 studies that reported the subsequent risk of PJI after superficial SSI in THAs, which normally would entail revision surgery. Both studies reported an increased risk of PJI after superficial SSI at 27.5% (11 PJIs out of 40 patients with superficial SSI in a cohort of 758 THAs) [3] and 50% respectively (4 PJIs out of 8 patients with superficial SSI in a cohort of 1,124 THAs) [6].

The low rate of superficial SSIs in our study compared with other studies [3,14,15] might be explained by a more aggressive treatment strategy at our institution, i.e., a low threshold for deep revision in patients with signs of SSI. This might in turn explain why we did not observe an increased revision risk after superficial SSI, as we might have been left with only true superficial SSIs.

### Limitations

This is a single-center study and thus the results may not be generalizable. Furthermore, this study is observational, which entails an increased risk of bias compared with a randomized controlled trial due to unmeasured confounding. However, because both superficial SSI after THA and revision hip arthroplasty are rare events [3,6,9,14], a randomized controlled study with the same research question would be very difficult to conduct.

Detailed information on relevant comorbidities such as diabetes mellitus, obesity, cardiovascular disease, or chronic kidney disease or on relevant lifestyle factors such as smoking or substance abuse was not available to us. However, to a certain extent such risk factors for both SSI and revision surgery are captured by the ASA score.

Unfortunately, the surveillance of SSIs does not include information on the length or type of antibiotic treatment. We do, however, know if patients with superficial SSI were treated surgically. Norway has a strict policy for antibiotic treatment in both the primary and specialist healthcare sector concerning the restriction of broad-spectrum antibiotics as well as using short courses of antibiotic treatment. We can say with confidence that patients with superficial SSI were not treated with antibiotic courses longer than 7–14 days at our institution and with reasonable confidence that the same holds true for the primary healthcare sector. In addition, we would argue that, even if some patients had received a longer treatment course, this would have had little chance to eradicate an overlooked deep SSI without surgical intervention and thus would most probably not have influenced our results.

Another limitation of this study is the small number of cases, i.e., patients who experienced a superficial SSI. Coupled with the relative rarity of the events, i.e., revision surgery, this accounts for the broad confidence intervals for the hazard ratios in the Cox models. The old adage “absence of evidence is not evidence of absence” comes to mind. However, here the Bayesian survival analysis, showing a low probability of an increased risk of subsequent revision surgery after superficial SSI, helps to put our results into context.

We note that adjustment for confounders had a larger influence on the Bayesian models. However, this is by no means a negative feature as, in accordance with our DAG, one would expect that adjusting for confounding would influence the association of the exposure with the primary endpoint and hence produce a more scientifically meaningful conclusion.

### Strengths

The data was obtained from a large cohort of patients who had undergone just 1 type of procedure, namely primary THA, while similar previous studies often included patients with both THA and total knee arthroplasties [3,6,14]. Furthermore, although this is a single-center study, revision surgeries performed at other hospitals are captured in the NAR. In addition, the completeness of reporting of revision surgery to the NAR and the follow-up for SSI by the local Department of Microbiology and Infection Control is high. Moreover, the fact that the same orthopedic surgeon has been consulted concerning equivocal cases of SSI during the whole study period contributes to good data quality.

### Conclusion

We showed no association between superficial SSI and revision after primary THA.

*In perspective*, while we do not advocate a lenient revision strategy concerning SSI, our data does not support the dogma that superficial SSI after primary THA does not exist.

## Notes

CP conceived the study and drafted the manuscript. OT performed the statistical analyses. All authors interpreted the results, contributed to the discussion, and reviewed the manuscript.

The authors would like to thank Anne Marie Fenstad for providing the relevant data from the NAR and Marita Skogstad and Sølvi Myrseth Westre for providing the SSI data.

Handling co-editors: Bart Pijls and Robin Christensen

*Acta* thanks Gary Hooper and Ewout S Veltman for help with peer review of this manuscript.
